# Progress in Medicine

**Published:** 1897-11-27

**Authors:** 


### Progress in Medicine.

### DISEASES OF THE DIGESTIVE ORGANS.

(*Continued from page* 99.)

**The Motor Activity of the Stomach.**—It is well known that the stomach has but small powers of absorption for many substances. Probably in man, as in some other animals, little, if any, water taken by the mouth passes through the lymphatics of the stomach. Meltzer[17] shows that when the pylorus and cardiac orifices are occluded no poisoning by strychnine takes place, even if large doses are given, but prussic acid is absorbed directly. Thus, in rabbits at least, absorption of strychnine takes place in the small intestine and not in the stomach. The same writer also investigated the manner of absorption from the peritoneal cavity. Of late it has been thought that this takes place through the blood-vessels, but he shows that small quantities at least are taken away by the lymphatics, and even large amounts often disappear through the same channels, but this varies with the individual. Even after death absorption takes place through the lymphatics.

The motor power of the stomach is undoubtedly the most important, but Meltzer finds that its contraction is chiefly carried out at the pyloric end, while at the outer end of the fundus an area exists where neither natural peristaltic waves nor contractions from direct or indirect electrical currents can be produced.[18] Thus the activity of the stomach is chiefly confined to the right half of the organ, and cicatrices or new growths in that portion produce effects quite different to those occurring near the cardiac end.

The phenomenon of merycism has been explained in various ways as a survival of true rumination in ancestors, or as a means of overcoming an excess of acid. Boas, however, found cases where the acid was deficient, and Einhorn one where the acidity was sometimes deficient and sometimes in excess. Treatment, the latter says, should include an analysis of the stomach contents. Acids or alkalies may then be given in accordance with the results. H. Miniassian[19] records a case of rumination where achlorydria existed with abnormally rapid passage of food through the stomach. He suggests that here the excessive motor activity of the stomach was the cause of the merycismus; at all events, the affection ceased when the patient was treated with hydrochloric acid after meals. It is curious, as Einhorn remarks, that the great majority of recorded cases, some 120, have been in males.

**Liver.**—In very rare cases an opening from the biliary passages into the lung has been formed, and bile brought up in the expectoration. Two such occurrences are reported by G. J. E. Graham,[20] who gives an account of those previously observed by Courvoisier and others, in all about 34, not reckoning those produced by tropical abscesses. The causes are either gallstones, hydatids, ascarides, or occasionally an injury. Recovery took place in about a third of the cases. Besides the expectoration of bile, we may find dulness over the right lung, extending up to the third intercostal space, with harsh breathing and large râles and violent cough. When from any cause there is an obstruction to the flow of bile through the lung jaundice quickly appears. When a calculus is present an abscess may have formed in the liver and ulcerated through the diaphragm and pleura into the lung, or an abscess may have formed around the gall-bladder and pushed its way above the liver into the lung direct.

Acute yellow atrophy in children is another rare affection, an instance of which is recorded by Cavafy,[20] the patient being only 12 years old. She was very jaundiced when she was admitted to St. George's Hospital, after about three weeks' illness. There was pyrexia, and the liver was at first enlarged; cerebral symptoms followed, and in a fortnight the patient sank into a comatose state, and from which she never recovered. The urine appeared free from leucin and tyrosin, the liver was largely replaced by fatty and granular *débris* and weighed 3 lb. 8 oz. Greves has collected some 17 cases in children, and a practical deduction is drawn by Cavafy from these instances that cases of apparently simple jaundice in children do sometimes terminate fatally, though presenting no special symptoms in the early stages. Fleet-Surgeon Griffiths[22] found the liver weighing only 28 ounces after 12 days' illness. The patient, an ordinary seaman, aged 19, suffered from vomiting, jaundice, and obstinate constipation, followed by unconsciousness and convulsions. W. Devereux chronicles[23] an instance of chronic cirrhosis with ascites, followed by the appearance of a large cyst of the liver. This was tapped, and a dark-brown fluid withdrawn containing much albumen, nucleated liver cells, albumen, and chlorides. A second drainage showed the absence of hydatid hooklets, cholesterin, or bile products, but abundant fats. Complete recovery followed. Simple cysts of the liver are, however, very rare, but it seemed possible to exclude pancreatic and splenic cysts, or malignant growths, as well as hydatids Atrophic cirrhosis may become quiescent after one or more removals of the ascites by tapping, but Merklen describes[24] a form which proves fatal in a few weeks or months, with œdema of the limbs and coma, with rapid wasting. He believes that it accompanies a fatty degeneration of the liver cells, and in a case described by him chylous ascites was also present without any lesion of the lymphatic ducts, cancer, or tubercle. He declares himself, however, quite ignorant of the manner in which a true chylous fluid is produced under these conditions.

The production of cirrhosis has been attributed to microbial action rendered possible by the chronic catarrh which alcohol causes in the intestinal tract. Ramond[25] tested this theory upon animals, and in one case, where alternate doses of alcohol and microbes had been given, found cirrhosis had occurred, though prolonged feeding on alcohol or on microbes ended in fatty degeneration. He believes that the alcohol in cirrhosis directly hinders the liver cells from keeping back the toxins, which thus affect the liver in turn. Hypertrophic cirrhosis with chronic jaundice would also seem to be a microbial disease, and is sometimes due to the bacillus coli which has gained access after an inflammatory attack, produced in the first instance by gallstones. Gilbert and Fournier[26] give the history of a patient where this could be clearly traced. The histological lesions were typical, and the bacillus was found both in the liver and spleen. In the former, it was also detected during life. Vaughan Harley[27] points out that when urobilin is found in the urine, it is clear that some bile reaches the intestine, for only there in the whole body is urobilin formed, whether jaundice be present or not. Again, as the liver tissue is destroyed in cirrhosis the urea is diminished, and its place is taken by ammonia. This may be used as a guide to diagnosis in the early stages, where we find it together with an increase of aromatic sulphates and indican. For treatment, the washing out of the stomach and even of the colon with boracic solution is indicated. Potassium iodide in 10 or 20 grain doses is of great value, and if there is active hyperæmia of the liver ammonium chloride and saline purgatives will give relief.

[17] Amer. Jour. Med. Sc., June. [18] N. Y. Med. Jour., April 24. [19] Med. Rec., June 12. [20] B. Med. Jour., June 5. [21] Lancet, July 17. [22] B. M. Jour., July 24. [23] Lancet, July 24. [24] Med. Week, June 4. [25] B. Med. Jour., July 17. [26] Med. Week, July 16. [27] Clinical Jour., July 21.

### OBSTETRICS.

(*Continued from page* 135.)

**Pelvic Hæmatocele.**—Cullingworth[43] under this heading places only those cases in which an intra-peritoneal effusion of blood occurs, usually behind, and displacing the uterus, and limited by encapsulation or adhesion. He does not include unlimited intra-peritoneal hæmorrhages or broad ligament hæmatomata. He considers that while unlimited intra-peritoneal hæmorrhage is usually due to rupture of the distended pregnant tube, that pelvic hæmatocele is due either to the escape of the ovum from the fimbriated extremity at an early stage of the affection, with hæmorrhage consequent on its detachment, or to effusion of blood into the tube, compression of the ovum, and formation of a tubal mole, with, at the same time, escape of blood from the abdominal extremity of the tube. He points out that the fimbriated extremity of the tube is usually open until the sixth week, and becomes occluded between the sixth and eighth weeks, after which hæmorrhage cannot take place except by rupture of the tube. He also shows that pelvic hæmatocele may be the result of rupture of the tube in cases where the lacerated vessels are either few or small and the resulting hæmorrhage slow. He gives the conditions found in 20 cases operated on by him, and found that every one was connected with tubal gestation, but that in only one had rupture of the tube occurred, and in one the hæmatocele, although found in a case in which tubal pregnancy existed, was caused by the rupture of a broad ligament cyst into which hæmorrhage had occurred, the pregnant tube being on the opposite side of the pelvis to the cyst. In 34 cases of early tubal gestation operated on, in 19 hæmorrhage from the free end of the tube was found to have taken place, with pelvic hæmatocele in 17, and free intra-peritoneal effusion in 2. In 9 cases where rupture had occurred, on the other hand, in 1 pelvic hæmatocele resulted, and in 1 a broad ligament hæmatoma; in the other 7 free intra-peritoneal hæmorrhage occurred. This shows clearly that pelvic hæmatocele is often associated with hæmorrhage from the free end of the tube without rupture, and also that rupture of the tube is not the necessary precursor of this condition. All his quoted cases are those in which the pathological conditions were verified by actual visual examination of the parts involved, and do not depend on clinical symptoms and vaginal examination only.

As guides for the withholding operative interference or not, he states that operative interference can only be safely withheld when the clinical symptoms point to the hæmorrhage having occurred within the early weeks of pregnancy; at a later stage than, say, the sixth week the risk of further hæmorrhage is too great for non-operative measures to be justifiable. If ever the symptoms are of sufficient severity to lead to suspicion of rupture operative interference is indicated, and is imperative if it is found that the swelling is increasing, or if repeated attacks of pain and faintness give reason to believe that hæmorrhage is still going on, while the same applies to all cases where the hæmorrhage is diffused in the peritoneal cavity.

Montgomery[44] advocates the treatment of these cases by vaginal incision and drainage, but this should not be attempted unless the evidence of hæmorrhage has ceased and encapsulation occurred. He advocates operative measures as preventing the retention of a large amount of possibly putrescible material in the pelvis, in close proximity to the rectum. An incision in the vaginal vault sufficient to admit two fingers is made, and the clots pressed and emptied out of the pelvic cavity; the cavity formed is then irrigated with normal saline solution. The tube and ovary on either side should be carefully examined, and if the tube on either side is found to be ruptured, or to contain a clot, it should be drawn into the vagina, ligatured, and removed. The pelvic cavity should then be packed with iodoform gauze for drainage. The incision is preferably made with the thermo-cautery, so as to prevent too rapid healing.

**Bicycling for Women.** — Thulaber[45] recommends cycling in cases of amenorrhœa, especially with undeveloped uterus. The contraindications appear to be, in his opinion, chronic or acute gonorrhœa, salpingitis, and subacute and chronic peritonitis of whatever origin. He advises against it if hæmorrhagic endometritis is present. It is inadmissible for patients with fibroid and ovarian tumours, and pregnancy is an absolute contraindication. It is not harmful in cases of flexion or version, and has produced good results in these conditions as well as in partial prolapse.

MacNaughton Jones[46] disapproves of cycling at the time of the menopause, especially if the patient is anæmic and has functional cardiac disturbance. He considers retro-displacements a contraindication to the exercise, especially in anæmic girls. With these reservations he approves highly of the exercise.

**Vesico-Vaginal Fistula.**—Stanmore Bishop[47] describes a new method of operating for this affection. He raises a circular flap of the vaginal mucous membrane around the fistula, the centre of the fistula being the centre of the circular incision. The diameter of the circle must be such that the flap when inverted will cover the opening, and leave a margin in excess. The flap of mucous membrane is dissected up until within a sixth of an inch of the edge of the fistula all around, two or more silk sutures are then passed through the circumference of the flap and drawn through the fistula into the bladder and out of the urethra; the flap is thus inverted into the bladder, so that its raw surface is in contact with itself, or rather forms a raw tube, while the outside of the tube, which is the surface in contact with the contents of the bladder, is covered by vaginal mucous membrane. The raw tube is then obliterated by two or three circular sutures, with the result that the fistula is closed by the inverted flap, which forms a small cylindrical or cylindriform projection on the floor of the bladder. The guiding threads by which the flap was inverted are then removed The advantages of the operation are that loss of time is avoided, and that the surface facing the bladder is covered with mucous membrane. It seems to promise great success in suitable cases.

[43] Lancet, June 19, 1897. [44] Clinical Journal, June 28, 1897, quoting from Annals of Gynæcology and Pediatry, June, 1897. [45] Amer. Journ. of Med. Sciences, March, 1897. [46] Amer. Journ. of Med. Sciences, loc. cit. [47] Lancet, June 19, 1897.

